# Further Characterization of Glycine-Containing Microcystins from the McMurdo Dry Valleys of Antarctica

**DOI:** 10.3390/toxins7020493

**Published:** 2015-02-10

**Authors:** Jonathan Puddick, Michèle R. Prinsep, Susanna A. Wood, Stephen Craig Cary, David P. Hamilton, Patrick T. Holland

**Affiliations:** 1 Cawthron Institute, Private Bag 2, Nelson 7010, New Zealand; E-Mails: susie.wood@cawthron.org.nz (S.A.W.); pth_consultancy@xtra.co.nz (P.T.H.); 2 Department of Chemistry, School of Science, University of Waikato, Private Bag 3105, Hamilton 3240, New Zealand; E-Mail: m.prinsep@waikato.ac.nz; 3 Environmental Research Institute, University of Waikato, Private Bag 3105, Hamilton 3240, New Zealand; E-Mail: d.hamilton@waikato.ac.nz; 4 Department of Biological Sciences, School of Science, University of Waikato, Private Bag 3105, Hamilton 3240, New Zealand; E-Mail: caryc@waikato.ac.nz

**Keywords:** microcystin, Antarctica, amino acid analysis, mass spectrometry, thiol derivatization

## Abstract

Microcystins are hepatotoxic cyclic peptides produced by several cyanobacterial genera worldwide. In 2008, our research group identified eight new glycine-containing microcystin congeners in two hydro-terrestrial mat samples from the McMurdo Dry Valleys of Eastern Antarctica. During the present study, high-resolution mass spectrometry, amino acid analysis and micro-scale thiol derivatization were used to further elucidate their structures. The Antarctic microcystin congeners contained the rare substitution of the position-1 d-alanine for glycine, as well as the acetyl desmethyl modification of the position-5 Adda moiety (3*S*-amino-9*S*-methoxy-2*S*,6,8*S*-trimethyl-10-phenyldeca-4*E*,6*E*-dienoic acid). Amino acid analysis was used to determine the stereochemistry of several of the amino acids and conclusively demonstrated the presence of glycine in the microcystins. A recently developed thiol derivatization technique showed that each microcystin contained dehydrobutyrine in position-7 instead of the commonly observed *N*-methyl dehydroalanine.

## 1. Introduction

The McMurdo Dry Valleys in Eastern Antarctica form the largest ice-free region in Antarctica and are characterized by low temperatures, minimal precipitation and strong winds [1]. Despite these harsh conditions, life is still present in this arid environment in the form of microbial communities [2,3,4]. Cyanobacteria proliferate in the moist areas in and around glacial streams and lakes and form thick benthic mats [5,6,7]. Many cyanobacteria genera around the world have been reported to produce hepatotoxic microcystins (MCs) [8], and this is also the case for cyanobacterial communities that grow in the harsh climates of the Arctic and Antarctica [9,10,11,12,13].

**Figure 1:**
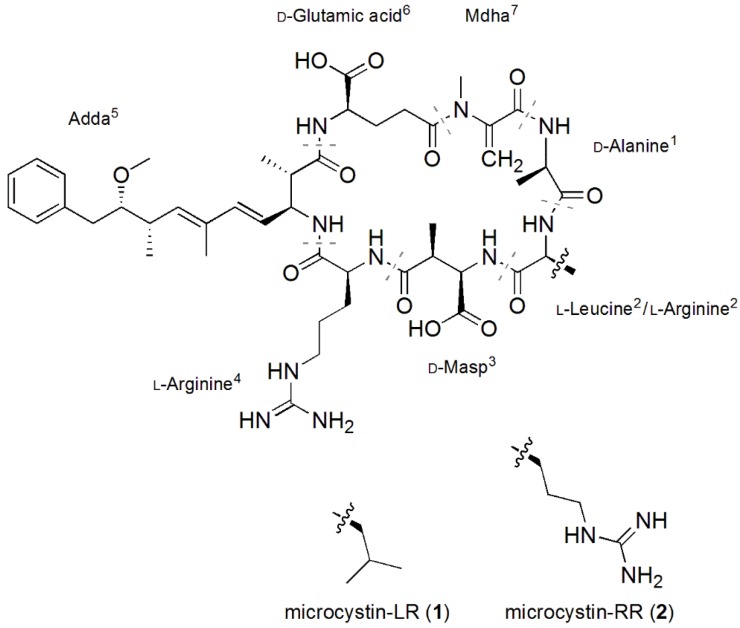
Structures of microcystin-LR (**1**) and microcystin-RR (**2**), where Adda is 3-amino-9-methoxy-2,6,8-trimethyl-10-phenyldeca-4,6-dienoic acid, Mdha is *N*-methyl dehydroalanine and Masp is methylaspartic acid.

Microcystins are a family of cyclic heptapeptides produced by a combination of non‑ribosomal peptide synthetase and polyketide synthase modules. As observed in MC-LR (**1**) and MC-RR (**2**; Figure 1), microcystins contain l-amino acids, d-amino acids and more unconventional amino acids, such as; Adda (3*S*-amino-9*S*-methoxy-2*S*,6,8*S*-trimethyl-10-phenyldeca-4*E*,6*E*-dienoic acid) or *N*-methyl dehydroalanine (Mdha). To date, there have been at least 100 different microcystin congeners characterized [14], mostly due to substitutions of the variable l-amino acids in positions-2 and -4, although modifications have been reported for all of the amino acids [15]. Substitution of the position-1 d-alanine is uncommon, and only substitutions for d-serine [16], d-leucine (Leu) [17,18] and methionine [19,20] have been reported to date.

In 2008, our research group showed that microcystin-producing cyanobacteria were particularly prolific in the McMurdo Dry Valleys of Antarctica. Each sample collected tested positive for at least low levels of microcystin [21]. Previously, only [Asp^3^] MC-LR, MC-LR and nodularin had been reported in Antarctic cyanobacteria [9,10,11], but our 2008 study also identified [Asp^3^, Dha^7^] MC-LR, MC-FR and MC-RR, including its [Asp^3^] and [Asp^3^, Dha^7^] congeners [21]. During the course of this study, a discrepancy between different methods of determining microcystin content was noted in several samples. Whilst a high concentration of microcystin was detected using an enzyme-linked immunosorbent assay and protein phosphatase inhibition assay, only low concentrations of some common microcystins were detected by liquid chromatography-tandem mass spectrometry (LC-MS/MS). Further investigation demonstrated that these samples contained eight new microcystins, which were not initially detected by the LC-MS/MS multiple reaction monitoring method.

The new microcystins contained several interesting structural modifications; the presence of homoarginine (Har) residues, acetylation of the Adda moiety (ADM Adda; acetyl desmethyl Adda) and substitution of the position one amino acid for glycine (Gly). The presence of these new microcystins was reported in the 2008 paper, and their structures were postulated based on the daughter ion spectra. Here, we present more in-depth characterization of these compounds and further clarification of their structures.

**Figure 2:**
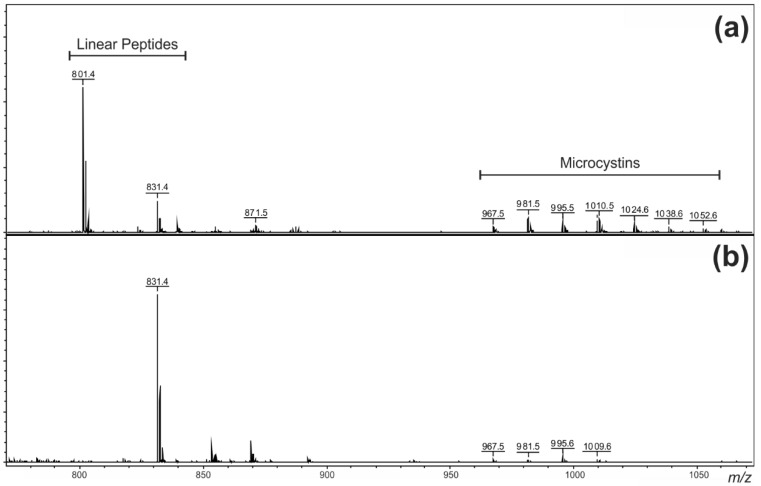
Positive ion matrix-assisted laser desorption/ionization-time of flight mass spectra of the two Miers Valley (Antarctica) samples of (**a**) MVAG1 and (**b**) MVMG1.

## 2. Results and Discussion

### 2.1. Oligopeptide Diversity in the Miers Valley Cyanobacterial Mats

Methanol extracts of two samples (Sample IDs: MVAG1 and MVMG1) collected from Miers Valley, Antarctica, were analysed by matrix-assisted laser desorption/ionization-time of flight (MALDI-TOF) MS. The positive ion mass spectra (Figure 2) and post-source decay (PSD) experiments indicated the presence of two groups of oligopeptides; six linear peptides with masses between 800 and 844 Da and eight microcystins between 966 and 1,051 Da. The linear peptides appear to consist of an ester-linked hydroxyphenyllactic acid *C*-terminus, two aromatic amino acids, isoleucine or leucine and an unusual 168-Da moiety at the *N*-terminus [22]. Further structural characterization of these new compounds remains a focus of our research group.

### 2.2. Structural Characterization of Eight Glycine-Containing Microcystins

**Figure 3:**
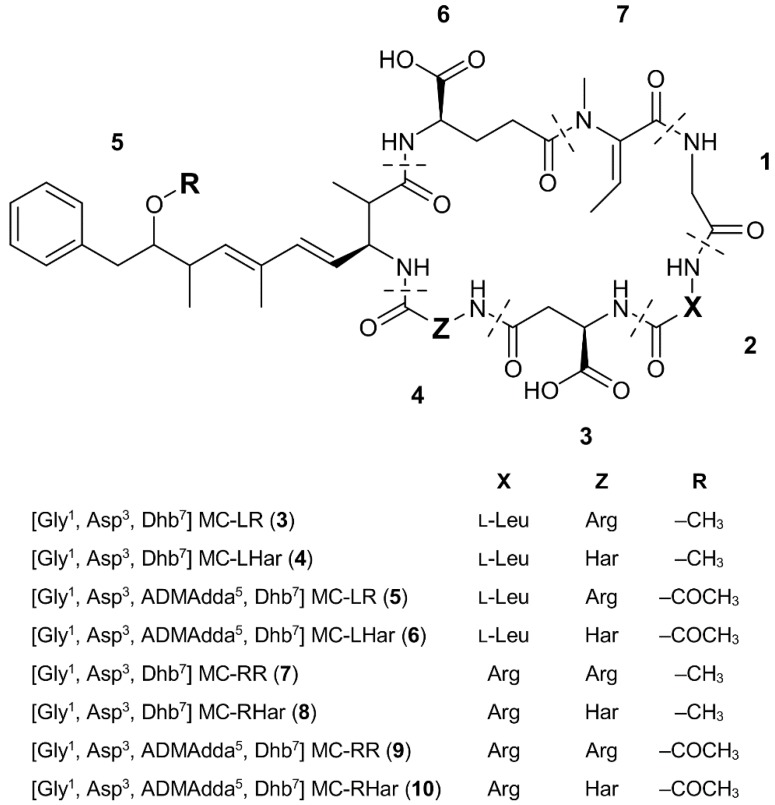
Structures of the eight Antarctic microcystin (MC) congeners (**3**–**10**) identified from two Miers Valley (Antarctica) samples; Arg, arginine; Dhb, dehydrobutyrine; Gly, glycine; Har, homoarginine; Leu, Leucine.

Analysis of the Miers Valley samples by LC-MS suggested that they contained four structural variants of MC-LR (referred to as Antarctic-LR congeners; Figure 3) and four variants of MC-RR (referred to as Antarctic-RR congeners), as these compounds were eluted from a reversed-phase C_18_ column within the appropriate retention regions (Table 1). Whilst the compounds possessed protonated ions that matched those of previously described microcystins, the MS/MS spectra indicated that all eight microcystins were new [21]. A combination of amino acid analysis, chemical derivatization and MS data was used to confirm the putative structures for **3**–**10**.

**Table 1:** Molecular masses and retention times of the eight Antarctic microcystin congeners (**3**–**10**) identified from two Miers Valley (Antarctica) samples and two common microcystin congeners (**1**–**2**).

Microcystin	M_r_^a^ (Da)	RT ^b^ (min)
MC-LR (**1**)	994.5	7.40
[Gly^1^, Asp^3^, Dhb^7^] MC-LR (**3**)	966.5	7.27
[Gly^1^, Asp^3^, Dhb^7^] MC-LHar (**4**)	980.5	7.34
[Gly^1^, Asp^3^, ADMAdda^5^, Dhb^7^] MC-LR (**5**)	994.5	7.29
[Gly^1^, Asp^3^, ADMAdda^5^, Dhb^7^] MC-LHar (**6**)	1008.5	7.36
MC-RR (**2**)	1023.6	6.48
[Gly^1^, Asp^3^, Dhb^7^] MC-RR (**7**)	1009.6	6.29
[Gly^1^, Asp^3^, Dhb^7^] MC-RHar (**8**)	1023.6	6.38
[Gly^1^, Asp^3^, ADMAdda^5^, Dhb^7^] MC-RR (**9**)	1037.6	6.33
[Gly^1^, Asp^3^, ADMAdda^5^, Dhb^7^] MC-RHar (**10**)	1051.6	6.42

^a^ Molecular weights are rounded to one decimal place; ^b^ RT = retention time on a C_18_ column as per [23].

As only a small amount of algal extract was available, fractionation of the new microcystins did not proceed beyond isolating two fractions containing a mixture of the four Antarctic-LR congeners and a mixture of the four Antarctic-RR congeners. High resolution mass spectrometry (HRMS) analysis was conducted on these semi-pure mixtures of the Antarctic microcystin congeners and gave mass-to-charge ratios consistent with the singly-protonated ions for structures **3**–**10** and mass deviations of less than 4 ppm (Supplementary Information Table S1). The accurate masses for **5**–**6** and **9**–**10** indicated that the 28-Da mass increase observed in the position-5 amino acid of these compounds was due to an additional carbonyl (ADMAdda), rather than two additional methyl groups.

Each mixture of four microcystins was also hydrolyzed and subjected to Advanced Marfey’s amino acid analysis [24,25] to determine the amino acids present and their stereochemistry. Liquid chromatography-MS analysis of the l-1-fluoro-2,4-dinitrophenyl-5-leucine (FDLA) derivatives of the hydrolysed Antarctic-LR congeners (Supplementary Information Figure S1) and comparison with standard amino acids (Supplementary Information Figure S2) confirmed the presence of d-aspartic acid (Asp; *m*/*z* 426; 12.9 min), d-glutamic acid (Glu; *m*/*z* 440; 14.3 min), Gly (*m*/*z* 368; 15.2 min) and l-Leu (*m*/*z* 424; 21.0 min). Comparison with the l-FDLA derivatives of hydrolysed MC-LR (Supplementary Information Figure S3) indicated the presence of 3(*S*)-Adda (*m*/*z* 592; 32.9 min) [25]. Amino acid analysis of the Antarctic-RR congeners revealed similar results (Supplementary Information Figure S4); the presence of d-Asp (*m*/*z* 426; 12.9 min), d-Glu (*m*/*z* 440; 14.3 min), Gly (*m*/*z* 368; 15.2 min) and 3(*S*)-Adda (*m*/*z* 592; 32.9 min).

**Figure 4:**
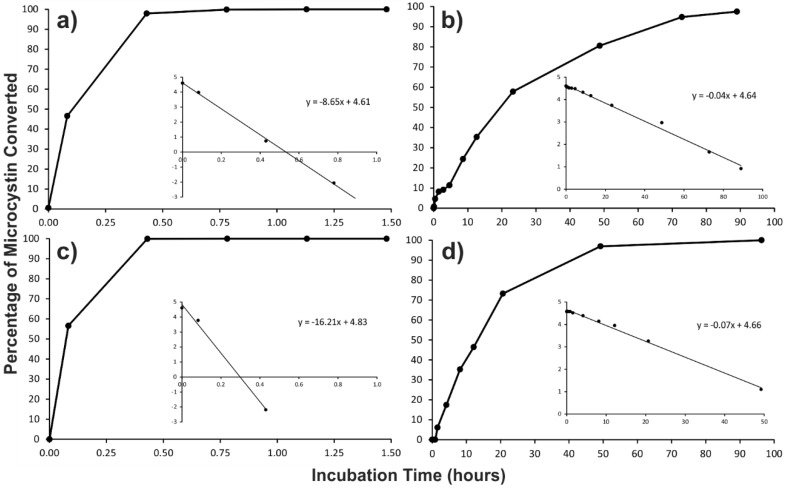
Time courses and first-order rate plots (inset) for the β-mercaptoethanol derivatization (at 30 °C; pH 9.7) of: (**a**) MC-LR; (**b**) the Antarctic-LR congeners identified in two Miers Valley (Antarctica) samples; (**c**) MC-RR; and (**d**) the Antarctic-RR congeners identified in two Miers Valley samples.

*N*-Methylamine (*m*/*z* 324; 19.6 min; Supplementary Information Figure S3) was not observed in the amino acid analysis of the Antarctic microcystin mixtures, but is commonly observed during microcystin analysis [25], as it is the product of the hydrolytic breakdown of Mdha. A micro-scale thiol derivatization was used to verify the absence of Mdha in the Antarctic microcystin congeners. A microcystin containing a terminal alkene, such as that in Mdha or dehydroalanine (Dha), will readily react with β‑mercaptoethanol under alkaline conditions [26,27]. Monitoring of the derivatization by LC-MS showed that MC-LR, which contains Mdha, reacted quickly with β-mercaptoethanol (*t*_½_ = 4.8 min; Figure 4a). A reaction rate that was approximately twice as fast was observed with a microcystin containing two arginine residues (MC-RR; *t*_½_ = 2.6 min; Figure 4c). However, with a microcystin containing dehydrobutyrine (Dhb), the reaction rate was hundreds of times slower [28]. When the Antarctic-LR congeners were derivatized with β-mercaptoethanol, the reaction rate was over two orders of magnitude slower than that of MC-LR (*t*_½_ = 1,089 min; Figure 4b). A similar difference in reaction rate was observed with the Antarctic-RR congeners when compared to MC-RR (*t*_½_ = 632 min; Figure 4d). The slow reaction rate with β-mercaptoethanol, in combination with the absence of *N*-methylamine in the amino acid analysis, gives a strong indication that the Antarctic microcystin congeners contained dehydrobutyrine (Dhb) instead of Mdha/Dha. This substitution could also be confirmed by analysis for the 2-ketobutyric acid produced from the hydrolytic breakdown of Dhb [29]; however, there was insufficient material to conduct these additional analyses. The presence of Dhb was unable to be confirmed during the 2008 study [21], when this amino acid was tentatively assigned as Mdha.

**Figure 5:**
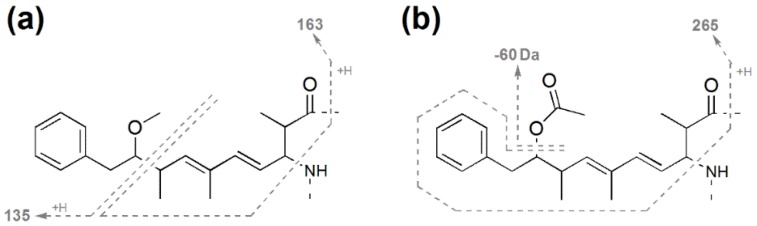
Tandem mass spectrometry fragments commonly observed in (**a**) Adda-containing microcystins and (**b**) in ADMAdda-containing microcystins.

**Figure 6:**
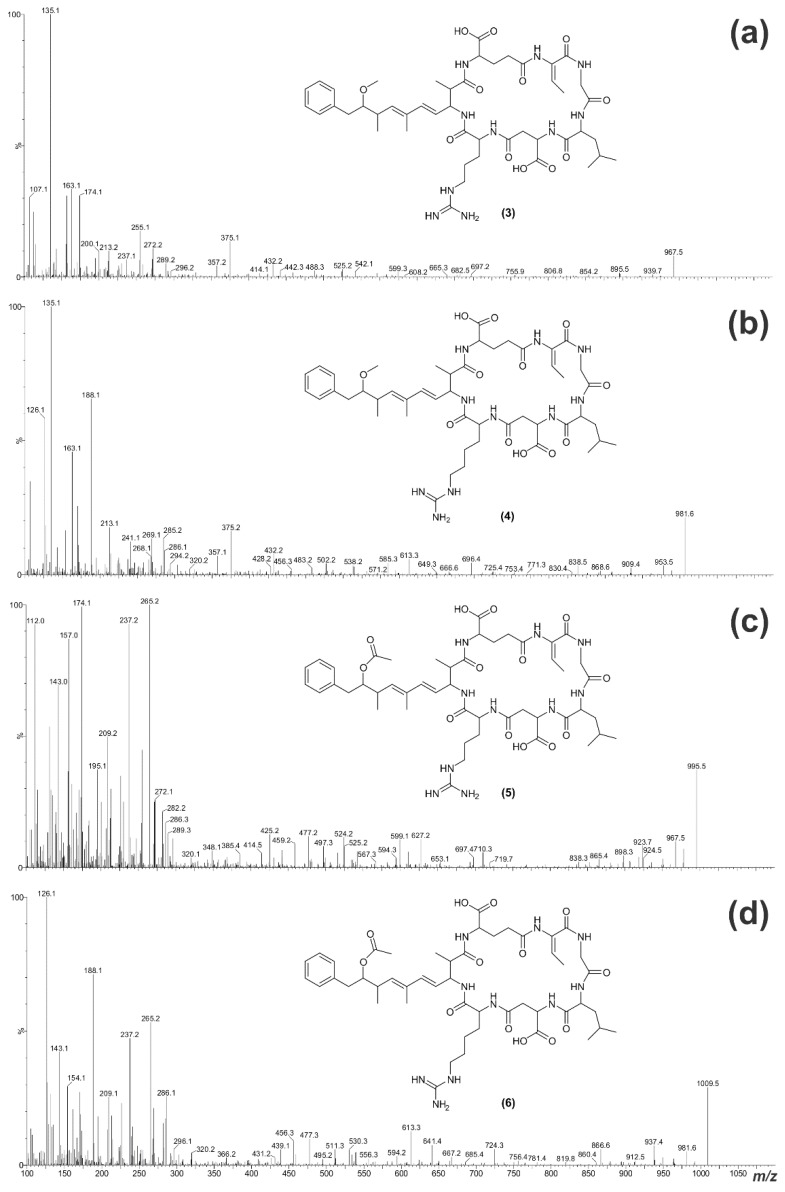
Electrospray ionization collision-induced dissociation tandem mass spectra for **3**–**6** (**a**–**d**) and the putative structures of the microcystins.

The Adda portion of the microcystin structure fragments under electrospray ionization collision-induced dissociation (ESI CID) conditions to form diagnostic ions (*m*/*z* 135 and 163; Figure 5a) commonly used for the identification and characterization of microcystins [30,31]. Microcystins containing the ADMAdda modification form different fragment ions under ESI CID conditions (*m*/*z* 60 and 265; Figure 5b) as the *O*-acetyl group dissociates from the main structure more readily [32].

The ESI CID MS/MS spectrum of **3** indicated that the compound contained Adda, as an intense *m*/*z* 135 fragment ion was present (Figure 6a) The *m*/*z* 112, 129 and 157 ions indicated that the microcystin contained an arginine (Arg) residue [33]. The later retention time on reversed-phase C_18_ (Table 1) suggested that it was unlikely that there were two Arg residues present. The mass difference between the fragment ions indicated the position of the remaining amino acids in the compound (Table 2).

**Table 2:** Tandem mass spectrometry fragment assignments for MC-LR (**1**) and **3**–**6** observed by electrospray ionization collision-induced dissociation.

Fragment Assignment ^a^	MC-LR ^b^ (1)	[Gly^1^, Asp^3^, Dhb^7^] MC-LR (3)	[Gly^1^, Asp^3^, Dhb^7^] MC-LHar (4)	[Gly^1^, Asp^3^, ADMAdda^5^, Dhb^7^] MC-LR (5)	[Gly^1^, Asp^3^, ADMAdda^5^, Dhb^7^] MC-LHar (6)
M + H	995.6	967.6	981.6	995.6	1,009.6
M − HOAc + H	-	-	-	935.5	949.3
(Me)Asp-Arg/Har-(ADM)Adda-Glu-Mdha/Dhb-Gly/Ala + H	882		868.6		896.9
Arg/Har-(ADM)Adda-Glu-Mdha/Dhb-Gly/Ala + H	753		753.3		781.4
(Me)Asp-Arg/Har-(ADM)Adda-Glu + H	728		728.4		756.4
Arg/Har-(ADM)Adda-Glu-Mdha/Dhb + H	682	682.5	696.1	710.3	724.3
Glu-Mdha/Dhb-Gly/Ala-Leu-(Me)Asp-Arg/Har + H	682		668.0		
Arg/Har-(ADM)Adda-Glu + H	599	599.3	613.2	627.2	641.4
(Me)Asp-Arg/Har-(ADM)Adda + H	599				627.3
(ADM)Adda’-Glu-Mdha/Dhb-Gly/Ala-Leu + H	559		545.4		
Mdha/Dhb-Gly/Ala-Leu-(Me)Asp-Arg/Har + H	553	525.2	539.4	525.2	539.3
Glu-Mdha/Dhb-Gly/Ala-Leu-(Me)Asp + H	526			498.2	
Arg/Har-(ADM)Adda + H	470				512.3
Gly/Ala-Leu-(Me)Asp-Arg/Har + H	470	442.3	456.2	442.1	456.1
(ADM)Adda’-Glu-Mdha/Dhb-Gly/Ala + H	446	432.2	432.1		
Leu-(Me)Asp-Arg/Har + H	399	385.1	399.1	385.4	
Glu-Mdha/Dhb-Gly/Ala-Leu + H	397			383.1	383.1
(ADM)Adda'-Glu-Mdha/Dhb + H	375	375.1	375.2	375.2	
Mdha/Dhb-Gly/Ala-Leu-(Me)Asp + H	397			369.2	
(ADM)Adda'-Glu + H	292	292.1	292.2	292.1	292.1
Gly/Ala-Leu-(Me)Asp + H	314		286.1	286.3	286.1
ADMAdda − HOAc + H	-	-	-	282.2	282.2
ADMAdda − HOAc − NH_3_ + H	-	-	-	265.1	265.2
(Me)Asp-Arg/Har + H	286	272.1	286.1	272.1	286.1
Glu-Mdha/Dhb-Gly/Ala + H	284	270.2	270.2	270.1	270.2
Mdha/Dhb-Gly/Ala-Leu + H	268	254.3	254.1	254.1	254.1
Leu-(Me)Asp + H	243		229.2	229.0	229.1
Glu-Mdha/Dhb + H	213	213.1	213.1	213.1	213.1
Gly/Ala-Leu + H	185	171.0	171.1	171.0	171.1
(ADM)Adda' + H	163	163.1	163.1	163.1	163.0
Arg/Har + H	157	157.0	171.1	157.1	171.1
Mdha/Dhb-Gly/Ala + H	155	141.0	141.0	141.1	141.1
(ADM)Adda sidechain	135	135.1	135.1	163.1	163.0
Arg/Har immonium	129	129.2	143.1	129.1	143.1
Arg/Har fragment	112	112.1	112.0/126.1	112.0	111.9/126.1

^a^ Fragments containing CO losses and NH_4_^+^ adducts were not included; Adda’ = Adda minus NH_2_ and the sidechain (C_9_H_11_O or C_10_H_11_O_2_); ^b^ *m*/*z* values for MC-LR fragments are theorized nominal values [34,35,36].

**Figure 7:**
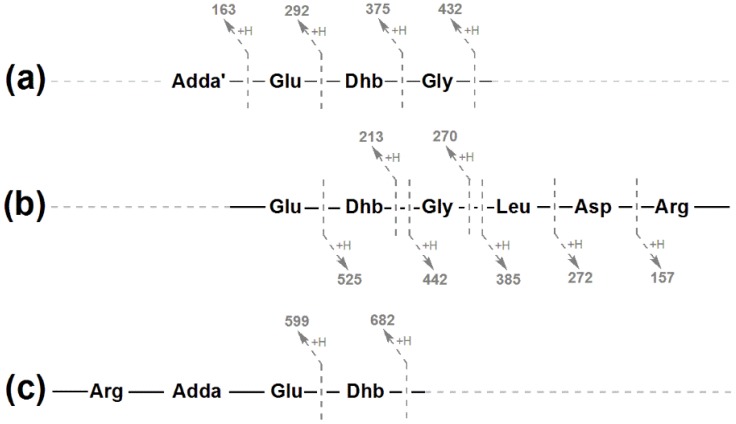
Tandem mass spectrometry fragment ions yielding three amino acid sub-sequences (**a**–**c**) which indicate the full amino acid sequence for [Gly^1^, Asp^3^, Dhb^7^] MC-LR (**3**).

The fragment ion series beginning with the Adda’ fragment (Adda minus NH_2_ and C_9_H_11_O; *m*/*z* 163; Figure 5a) indicated that **3** contained Glu and Dhb in positions six and seven, respectively (Figure 7a). The *m*/*z* 432 ion extended this ion series to include a Gly in position-1, yielding a sequence of Adda-Glu-Dhb-Gly. Another fragment ion series, which began with Arg (*m*/*z* 157), extended to include Asp, Leu, Gly, Dhb and Glu (Figure 7b). This gave a sequence of Arg-Asp-Leu-Gly-Dhb-Glu, the end of which overlapped with the previous sequence, resulting in a complete peptide sequence of Adda-Glu-Dhb-Gly-Leu-Asp-Arg. The *m*/*z* 599 ion (Arg-Adda-Glu) indicated that the Arg was joined to Adda and that the structure was cyclic (Figure 7c). The *m*/*z* 126, 143 and 171 ions in the MS/MS spectrum for **4** (Figure 6b) suggested that the microcystin contained a homoarginine (Har). Assignment of the daughter ions (Table 2) placed the Har residue in position-4.

The MS/MS spectrum for **5** (Figure 6c) did not contain an intense *m*/*z* 135 fragment ion. However, a loss of 60 Da (HOAc) was evident, which indicated that **5** contained an *O*-acetyl group. The *m*/*z* 265 ion suggested that this was due to an *O*-acetyl group on the Adda moiety (ADMAdda; Figure 4b) [37]. As with **3**, it was likely that a single Arg residue was present in this microcystin. Comparison of the MS/MS spectrum with that of **3** indicated that much of the structure for **5** was the same (Table 2), apart from the inclusion of ADMAdda in position-5. The MS/MS spectrum for **6** (Figure 6d) indicated that the structure was similar to **5**, except that the position-4 amino acid was Har.

**Figure 8:**
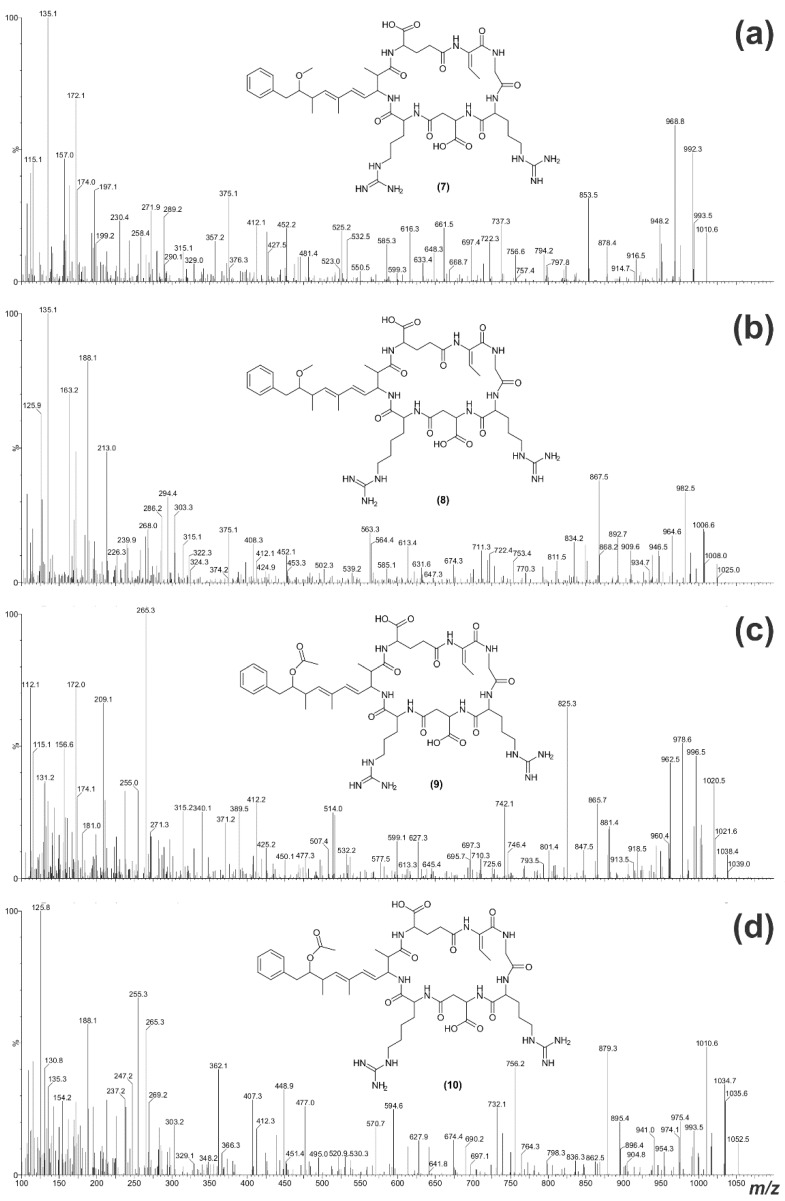
Electrospray ionization collision-induced dissociation tandem mass spectra for **7**–**10** (**a**–**d**) and the putative structures of the microcystins.

The daughter ion spectrum for **7** (Figure 8a) contained an intense *m*/*z* 135 fragment ion, suggesting the presence of Adda. The *m*/*z* 112, 129 and 157 ions in the spectrum indicated that **7** contained an Arg residue. However, the earlier retention time on C_18_ (Table 1) and loss of 42 Da (CN_2_H_2_) in the MS/MS spectrum suggested that there were two Arg residues present [35]. Comparison with the MS/MS spectrum for **3** indicated that much of the structure was very similar, except that the Leu at position-2 had been replaced with Arg (Table 3). The fragment ions for **8** (Figure 8b) showed that the compound was similar to **7**, except that the position-4 amino acid was Har.

The MS/MS spectrum for **9** did not contain an intense *m*/*z* 135 fragment ion, but the presence of ADMAdda was suggested by the *m*/*z* 265 fragment and a loss of 60 Da (Figure 8c). As with **7**, the inclusion of two Arg residues was indicated by diagnostic ions (*m*/*z* 112, 129 and 157) and the earlier retention time on reversed-phase C_18_ (Table 1). Comparison with the MS/MS spectrum for **7** indicated that much of the structure for **9** was the same, apart from the inclusion of ADMAdda at position-5 (Table 3). Likewise, **10** (Figure 8d) was structurally similar to **9**, except that Har was present in position-4.

**Table 3:** Tandem mass spectrometry fragment assignments for MC-RR (**2**) and **7**–**10** observed by electrospray ionization collision-induced dissociation.

Fragment Assignment ^a^	MC-RR ^b^ (2)	[Gly^1^, Asp^3^, Dhb^7^] MC-RR (7)	[Gly^1^, Asp^3^, Dhb^7^] MC-RHar (8)	[Gly^1^, Asp^3^, ADMAdda^5^, Dhb^7^] MC-RR (9)	[Gly^1^, Asp^3^, ADMAdda^5^, Dhb^7^] MC-RHar (10)
M + H	1024.7	1010.7	1024.7	1038.7	1052.7
M − CN_2_H_2_ + H	982	968.9	982.5	996.5	1010.8
M − HOAc + H	-	-	-	978.6	992.5
Arg/Har-(ADM)Adda-Glu-Mdha/Dhb-Gly/Ala-Arg + H	909	895.3	909.6		
(Me)Asp-Arg/Har-(ADM)Adda-Glu-Mdha/Dhb-Gly/Ala + H	882		868.6		896.4
(Me)Asp-Arg/Har-(ADM)Adda-Glu-Mdha/Dhb + H	811		811.5	825.1	839.8
Arg/Har-(ADM)Adda-Glu-Mdha/Dhb-Gly/Ala + H	753	739.2	753.4		781.0
(Me)Asp-Arg/Har-(ADM)Adda-Glu + H	728	714.3	728.3	742.1	756.2
Arg/Har-(ADM)Adda-Glu-Mdha/Dhb + H	682	682.2		710.2	724.2
Glu-Mdha/Dhb-Gly/Ala-Arg-(Me)Asp-Arg + H	725	697.5		697.3	
Arg/Har-(ADM)Adda-Glu + H	599		613.5		641.2
(Me)Asp-Arg/Har-(ADM)Adda + H	599	585.2	599.2	613.4	627.9
Mdha/Dhb-Gly/Ala-Arg-(Me)Asp-Arg/Har + H	596		582.4	568.7	
Har-ADMAdda − HOAC + H	-	-	-		452.5
(ADM)Adda'-Glu-Mdha/Dhb-Gly/Ala + H	446	432.2			
Glu-Mdha/Dhb-Gly/Ala-Arg + H	440	426.4		426.2	
Mdha/Dhb-Gly/Ala-Arg-(Me)Asp + H	440	412.1	412.3	412.2	412.3
(ADM)Adda'-Glu-Mdha/Dhb + H	375	375.1	375.2		
Gly/Ala-Arg-(Me)Asp + H	357	329.1		329.1	329.1
Mdha/Dhb-Gly/Ala-Arg + H	311	297.1	296.9	297.0	297.0
(ADM)Adda'-Glu + H	292			292.1	
ADMAdda − HOAc + H	-	-	-	282.3	282.1
ADMAdda − HOAc − NH_3_ + H	-	-	-	265.4	265.3
(Me)Asp-Arg/Har + H	286	272.3	286.2		286.1
Arg-(Me)Asp + H	286	272.3	272.1	272.2	272.5
Glu-Mdha/Dhb-Gly/Ala + H	284	270.0		270.1	
Gly/Ala-Arg + H	228	214.1	214.0	214.1	214.2
Glu-Mdha/Dhb + H	213	213.1	212.9	213.1	213.2
(ADM)Adda' + H	163	163.0	162.9	163.0	
Har + H	-	-		-	171.0
Arg + H	157	157.0	157.1	156.6	156.9
Mdha/Dhb-Gly/Ala + H	155	141.5	140.9	141.2	140.7
(ADM)Adda sidechain	135	135.1	135.1	163.0	
Har immonium	-	-	143.1	-	143.1
Arg immonium	129	129.5	129.1	129.1	
Har fragment	-	-	112.0/126.0	-	111.8/125.8
Arg fragment	112	112.2	112.0	112.1	111.8

^a^ Fragments containing CO losses and NH_4_^+^ adducts were not included; Adda’ = Adda minus NH_2_ and the sidechain (C_9_H_11_O or C_10_H_11_O_2_); ^b^ *m*/*z* values for MC-RR fragments are theorized nominal values [31,35].

The LC-MS/MS assignments for the Antarctic microcystin congeners were consistent with previous analyses of low‑energy, collision-activated spectra from similar microcystin variants [32,38,39,40]. Whilst the glycine substitution in position-1 is novel, the fragment ion series observed were similar to those reported for other microcystin congeners containing a position-1 substitution [16,17]. In several of the spectra, low-intensity *m*/*z* 155 (Mdha/Dhb-Ala) and *m*/*z* 135 (Adda sidechain fragment) ions were present along with the predominant ADMAdda congener ions. This was possibly due to the presence of small amounts of MC-LR and MC-RR in the concentrated extracts, as the contaminant ions were present at much lower intensities than would be expected. Furthermore, in each of the spectra containing these ions, the contaminant ion series do not continue further than these two easily-formed low‑mass ions.

Each of the new microcystins contained a d-Asp in position-3, which has been frequently observed in multiple cyanobacterial genera, including *Anabaena* [41], *Microcystis* [30], *Nostoc* [42], *Oscillatoria* [43] and *Planktothrix* [44], as well as cyanobacteria from Antarctica [11]. The position-7 Dhb has been reported in at least 15 other microcystins [43,44,45,46,47,48,49], three of which were ADMAdda-containing microcystins [46]. However, the frequency of the occurrence of Dhb-containing microcystins is potentially underestimated, as many microcystin congeners have been characterized solely by MS/MS. This does not allow for discrimination between the isometric Mdha and Dhb. The micro-scale thiol derivatization [28] utilized in this work will be of great utility to confirm the identity of the position-7 amino acid in microcystins where the sample size is limited.

The position-1 alanine in microcystins is highly conserved, but substitutions for leucine, serine and methionine have been reported [16,17,18,19,20]. However, prior to our 2008 study [21], a substitution for glycine had not been observed, although the adenylation domain responsible for incorporating the position-1 amino acid in microcystins (McyA2) shows structural similarity to the saframycin synthetase glycine adenylation domain [50,51].

Substitution of Arg for Har is rare, with only five microcystin congeners containing this amino acid being characterized to date [35,42,48,52,53]. Two further microcystins have been identified as containing Har, but full structures have not been reported [38]. At least thirteen ADMAdda-containing microcystins have been reported [16,37,39,42,46,52,54] from *Nostoc* and *Planktothrix* species from across Europe. Although the cyanobacterial strain responsible for the production of the new Antarctic microcystins was not isolated and cultured, molecular investigations identified the microcystin-producing cyanobacterium to be of the genus *Nostoc* [21].

The MS/MS analysis indicated the presence of Arg in six of the microcystins and Har in four of the microcystins. The amino acid analysis protocol used had poor sensitivity for arginine-like residues, and with the small sample size available, these amino acids were not detected. The MS/MS analysis also suggested that four of the microcystins contained ADMAdda. It is highly probable that ADMAdda would lose the *O*-acetyl group in the same manner as the *O*-methyl group of Adda is lost during acid hydrolysis [25,46]; hence, the two moieties would form the same hydroxylated product.

The small sample size available (<50 µg of each congener) prevented purification of the Antarctic microcystin congeners from proceeding beyond the separation of two mixtures containing the Antarctic‑LR congeners and the Antarctic‑RR congeners from the other components in the extract. Therefore, no bioactivity screening was conducted. Other microcystins containing similar modifications to these new congeners have been shown to be relatively potent in the mouse bioassay [46,52].

The identification of eight microcystin congeners containing uncommon modifications (glycine in position-1, homoarginine residues and ADMAdda modifications) was a significant discovery when first reported in 2008 [21]. At the time, it was the first report of ADMAdda-containing microcystins from the Southern Hemisphere, and to the best of our knowledge, these are still the only microcystins reported that contain glycine. In the present paper, these structures have been further clarified using additional analyses that have identified position-7 Dhb moieties in each of the microcystins and determined the stereochemistry of several of the amino acids. Although every effort was made to gather as much structural information as possible on these new microcystins, the small amount of available material precluded purification of individual congeners and nuclear magnetic resonance studies. However, the MS data reported here and the amino acid analyses are consistent with the reported structures.

## 3. Experimental Section

### 3.1. General Experimental Procedures

MALDI-TOF measurements were made on a AutoFlex II mass spectrometer (Bruker, Ballerica, MA, USA). LC-MS and LC‑MS/MS analyses were performed on a Bruker AmaZon X ESI ion-trap mass spectrometer coupled to an UltiMate 3000 HPLC system (Dionex, Sunnyvale, CA, USA) or a Quattro Ultima TSQ triple-quadrupole mass spectrometer (Waters-Micromass, Manchester, UK) coupled to a Waters Alliance 2695 HPLC system (Waters Corporation, Millford, DE, USA). HRESIMS was performed on a Bruker MicrOTOF mass spectrometer Reversed-phased C_18_ separations were conducted using YMC‑gel ODS-A (YMC Corporation, Kyoto, Japan). HPLC purification was performed using Waters 515 HPLC pumps (Waters Corporation, Millford, DE, USA) coupled to a Waters 2996 photodiode array detector (200–400 nm); and a Luna C_18_ column (150 × 4.6 mm, 5-μm; Phenomenex, Torrance, CA, USA).

### 3.2. Sample Collection

In December, 2006, two hydro-terrestrial cyanobacterial mat samples were collected from Miers Valley in the McMurdo Dry Valleys, Antarctica. These samples were obtained from the moist areas in front of Adams Glacier (MVAG1; 78°6'36''S, 163°54'20''E) and Miers Glacier (MVMG1; 78°5'42''S, 163°55'38''E). Microbial mat material was collected with a stainless steel spatula (swabbed with EtOH between samples) and placed in sterile 50 mL Falcon tubes. Samples were stored in the dark, below 0 °C in the field and at −80 °C in the laboratory until analysed. Vouchers of MVAG1 and MVMG1 are retained at the Cawthron Institute (Nelson, New Zealand).

### 3.3. Matrix-Assisted Laser Desorption/Ionization-Time of Flight Mass Spectrometry Analysis

Sample extracts were analysed by MALDI-TOF MS and MALDI PSD as described in Puddick *et al.*, 2014 [55], using α-cyano-4-hydroxycinnamic acid as a matrix.

### 3.4. Liquid Chromatography-Mass Spectrometry Analyses

Tandem mass spectrometry analyses of the microcystin samples were conducted on a Waters-Micromass Quattro Ultima TSQ mass spectrometer (Waters-Micromass, Manchester, UK), as described in Wood *et al.*, 2008 [21]. Thiol derivatization reactions were conducted on a Bruker AmaZon X mass spectrometer as described in Puddick *et al.*, 2013 [23].

### 3.5. Isolation of Semi-Pure Mixtures of the Antarctic Microcystins

Following completion of the 2008 analyses [21], the remaining material of the MVAG1 and MVMG1 samples was fractionated in order to undertake amino acid analysis and HRMS. MVAG1 (21 g dry weight) and MVMG1 (1 g dry weight) were extracted in 70% MeOH (300 mL) by disrupting the cells using an ultrasonic bath (35 W; 30 min). After vacuum filtration (#1 filter paper), the remaining cell material was re-extracted and filtered four more times. The resulting extract was gravity filtered, concentrated under vacuum and dried at 35 °C under a flow of nitrogen.

The crude extract (51.2 mg) was fractionated by reversed-phase C_18_ column chromatography (20 g) using a steep stepped gradient from water to MeOH to DCM, where **3**–**10** were eluted between 7:3 and 9:1 MeOH/H_2_O. Fractions containing **3**–**10** were combined (4.1 mg) and separated on a reversed-phase C_18_ column (20 g) using a stepped gradient from water to MeOH, where **3**–**6** were eluted with 1:1 MeOH/H_2_O and **7**–**10** with 3:2 MeOH/H_2_O.

The fraction containing **3**–**6** (2 mg) was subjected to HPLC using a gradient of water + 0.05% trifluoroacetic acid (TFA; A) to ACN + 0.05% TFA (B) at 1 mL/min. The sample was loaded in 10% B, which increased to 30% B over 3 min and then to 60% B over the subsequent 13 min, before washing with 100% B and re-equilibrating with 10% B. This yielded a mixture of the four Antarctic-LR congeners (16 min; **3**–**6**; ≤0.2 mg). The fraction containing **7**–**10** (1.2 mg) was similarly fractionated by HPLC. The sample was loaded in 10% B, which increased to 55% B over 16 min, before washing with 100% B and re-equilibrating with 10% B. This yielded a mixture of the four Antarctic‑RR (17 min; **7**–**10**; ≤0.2 mg).

### 3.6. Advanced Marfey’s Amino Acid Analysis

Semi-pure mixtures of the four Antarctic-LR congeners and the four Antarctic-RR congeners were subjected to amino acid analysis according to the advanced Marfey’s method [24,25]. 1-Fluoro-2,4-dinitrophenyl-5-leucine (FDLA) was synthesized according to the method of Marfey [56], but using leucinamide (Bachem, Bubendorf, Switzerland) instead of alaninamide. Both the d- and l- forms of the reagent were synthesized from the respective stereoisomers of leucinamide. Microcystin mixtures (≤200 µg) were prepared, derivatized and analysed as described in Puddick *et al.*, 2013 [57]. Samples were hydrolysed in 6 N HCl (0.5 mL) at 110 °C for 16 h. HCl was removed by drying before the hydrolysates were resuspended in H_2_O (105 µL) and divided into two aliquots (50 µL each). After pH adjustment with 1 M NaHCO_3_ (20 µL), 1% l- or dl-FDLA (*w*/*v*; 100 µL) was added, and the samples were incubated at 40 °C for 1 h. After being quenched with 1 N HCl (20 µL), the derivatized amino acids were diluted with MeOH (810 µL), centrifuged (14,000× *g*, 5 min) and analysed by LC-MS using an Econosil C_18_ column (250 × 3.2 mm, 5‑µ; Alltech, Deerfield, United States of America). Eluting derivatives were detected by UV absorption (250–500 nm) and ESI MS (negative ion mode, *m*/*z* 300–1100). The retention times of the l-FDLA derivatives of standard amino acids were as follows: l-Asp (12.2 min), l-Glu (13.5 min), Gly (15.2 min), l-Leu (21.0 min). The retention times of the d-FDLA derivatives of standard amino acids were as follows: l-Asp (12.9 min), l-Glu (14.3 min), Gly (15.2 min), l-Leu (26.7 min). The retention times of the l-FDLA derivatives were as follows: **3**–**6**: d-Asp (12.9 min), d-Glu (14.3 min), Gly (15.2 min), l-Leu (21.0 min), 3(*S*)-Adda (32.9 min); **7**–**10**: d-Asp (12.9 min), d-Glu (14.3 min), Gly (15.2 min), 3(*S*)-Adda (32.9 min).

### 3.7. β-Mercaptoethanol Derivatization for Mdha/Dhb Determination

A thiol derivatization technique [28] was used to determine which of the isometric amino acids, Mdha or Dhb, was present in the Antarctic microcystins. Standard microcystins (MC-LR and MC-RR) or semi-pure mixtures of the Antarctic microcystin congeners were dissolved in methanol (1.42 mL), mixed with 200 mM NaHCO_3_ (pH 9.7; 360 µL) in a septum-capped vial and left to equilibrate at 30 °C. Following LC-MS analysis of the original sample, β-mercaptoethanol (20 µL) was added and the vial inverted to mix. The reaction mixture was maintained at 30 °C in the sample tray of the LC-MS, and injections were made periodically over a 96-h period.

## 4. Conclusions

A cyanobacterial mat sample from Miers Valley in Antarctica was investigated for the presence of new oligopeptides. The putative structures of eight microcystins (**3**–**10**) containing a position-1 glycine were further characterized using a combination of amino acid analysis, chemical derivatization and MS/MS. The presence of the rare substitution of the position-1 amino acid for glycine was confirmed using amino acid analysis, as was the stereochemistry of several other structural elements (l-Leu, d-Glu, d-Asp and 3(*S*)-Adda). Tandem MS indicated the presence of Har and ADMAdda residues, which are uncommon modifications in microcystins. Amino acid analysis and thiol derivatization indicated that the position-7 amino acid was Dhb and not Mdha, which is commonly observed in microcystins. The micro-scale thiol derivatization technique [28] was invaluable for confirming the identity of the position-7 amino acid when dealing with such a small quantity of sample.

## Supplementary Materials

Supplementary materials can be accessed at: http://www.mdpi.com/2072-6651/7/2/0493/s1.

## References

[1] Doran P.T., McKay C.P., Clow G.D., Dana G.L., Fountain A.G., Nylen T., Lyons W.B. (2002). Valley floor climate observations from the McMurdo dry valleys, Antarctica, 1986–2000. J. Geophys. Res..

[2] Cowan D., Russell N., Mamais A., Sheppard D. (2002). Antarctic Dry Valley mineral soils contain unexpectedly high levels of microbial biomass. Extremophiles.

[3] Wynn-Williams D.D. (1990). Ecological aspects of Antarctic microbiology. Adv. Microb. Ecol..

[4] Vishniac H.S., Friedmann I.E. (1993). The microbiology of Antarctic soils. Antarctic microbiology.

[5] Taton A., Grubisic S., Balthasart P., Hodgson D.A., Laybourn-Parry J., Wilmotte A. (2006). Biogeographical distribution and ecological ranges of benthic cyanobacteria in east Antarctic lakes. FEMS Microbiol. Ecol..

[6] Cavacini P. (2001). Soil algae from northern Victoria Land (Antarctica). Polar Biosci..

[7] Fumanti B., Cavacini P., Alfinito S. (1996). Benthic algal mats of some lakes of Inexpressible Island (northern Victoria Land, Antarctica). Polar Biol..

[8] Sivonen K., Jones G., Chorus I., Bartram J. (1999). Cyanobacterial toxins. Toxic Cyanobacteria in Water: A Guide to Their Public Health Consequences, Monitoring and Management.

[9] Hitzfeld B.C., Lampert C.S., Spaeth N., Mountfort D., Kaspar H., Dietrich D.R. (2000). Toxin production in cyanobacterial mats from ponds on the McMurdo Ice Shelf, Antarctica. Toxicon.

[10] Jungblut A.-D., Hawes I., Mountfort D., Hitzfeld B., Dietrich D.R., Burns B.P., Neilan B.A. (2005). Diversity within cyanobacterial mat communities in variable salinity meltwater ponds of McMurdo Ice Shelf, Antarctica. Environ. Microbiol..

[11] Jungblut A.-D., Hoeger S.J., Mountfort D., Hitzfeld B.C., Dietrich D.R., Neilan B.A. (2006). Characterization of microcystin production in an Antarctic cyanobacterial mat community. Toxicon.

[12] Kleinteich J., Wood S.A., Puddick J., Schleheck D., Kupper F.C., Dietrich D. (2013). Potent toxins in Arctic environments—Presence of saxitoxins and an unusual microcystin variant in Arctic freshwater ecosystems. Chem. Biol. Interact..

[13] Kleinteich J., Wood S.A., Kupper F.C., Camacho A., Quesada A., Frickey T., Dietrich D.R. (2012). Temperature-related changes in polar cyanobacterial mat diversity and toxin production. Nat. Clim. Chang..

[14] Niedermeyer T. Microcystin congeners described in the literature. http://dx.doi.org/10.6084/m9.figshare.880756.

[15] Rinehart K., Namikoshi M., Choi B. (1994). Structure and biosynthesis of toxins from blue-green algae (*cyanobacteria*). J. Appl. Phycol..

[16] Sivonen K., Namikoshi M., Evans W.R., Fardig M., Carmichael W.W., Rinehart K.L. (1992). Three new microcystins, cyclic heptapeptide hepatotoxins, from *Nostoc* sp. strain 152. Chem. Res. Toxicol..

[17] Park H., Namikoshi M., Brittain S.M., Carmichael W.W., Murphy T. (2001). [D-Leu^1^] microcystin-LR, a new microcystin isolated from waterbloom in a Canadian prairie lake. Toxicon.

[18] Matthiensen A., Beattie K.A., Yunes J.S., Kaya K., Codd G.A. (2000). [D-Leu^1^]Microcystin-LR, from the cyanobacterium *Microcystis* RST 9501 and from a *Microcystis* bloom in the Patos Lagoon estuary, Brazil. Phytochemistry.

[19] Shishido T.K., Kaasalainen U., Fewer D.P., Rouhiainen L., Jokela J., Wahlsten M., Fiore M.F., Yunes J.S., Rikkinen J., Sivonen K. (2013). Convergent evolution of [D-Leucine^1^] microcystin-LR in taxonomically disparate cyanobacteria. BMC Evol. Biol..

[20] Qi Y., Rosso L., Sedan D., Giannuzzi L., Andrinolo D., Volmer D.A. (2015). Seven new microcystin variants discovered from a native *Microcystis aeruginosa* strain–unambiguous assignment of product ions by tandem mass spectrometry. Rapid Commun. Mass Spectrom..

[21] Wood S.A., Mountfort D., Selwood A.I., Holland P.T., Puddick J., Cary S.C. (2008). Widespread distribution and identification of eight novel microcystins in Antarctic cyanobacterial mats. Appl. Environ. Microbiol..

[22] Puddick J. (2013). Spectroscopic investigations of oligopeptides from aquatic cyanobacteria: Characterisation of new oligopeptides, development of microcystin quantification tools and investigations into microcystin production. Ph.D. Thesis.

[23] Puddick J., Prinsep M.R., Wood S.A., Miles C.O., Rise F., Cary S.C., Hamilton D.P., Wilkins A.L. (2013). Structural characterization of new microcystins containing tryptophan and oxidized tryptophan residues. Mar. Drugs.

[24] Fujii K., Ikai Y., Mayumi T., Oka H., Suzuki M., Harada K.-I. (1997). A nonempirical method using LC/MS for determination of the absolute configuration of constituent amino acids in a peptide: Elucidation of limitations of Marfey's method and of its separation mechanism. Anal. Chem..

[25] Fujii K., Ikai Y., Oka H., Suzuki M., Harada K.-I. (1997). A nonempirical method using LC/MS for determination of the absolute configuration of constituent amino acids in a peptide: Combination of Marfey’s method with mass spectrometry and its practical application. Anal. Chem..

[26] Miles C.O., Sandvik M., Nonga H.E., Rundberget T., Wilkins A.L., Rise F., Ballot A. (2012). Thiol derivatization for LC-MS identification of microcystins in complex matrices. Environ. Toxicol..

[27] Smith J.L., Boyer G.L. (2009). Standardization of microcystin extraction from fish tissues: A novel internal standard as a surrogate for polar and non-polar variants. Toxicon.

[28] Miles C.O., Sandvik M., Haande S., Nonga H., Ballot A. (2013). First use of LC-MS analysis with thiol derivatization to differentiate [Dhb^7^]- from [Mdha^7^]-microcystins: Analysis of cyanobacterial blooms, *Planktothrix* cultures and European crayfish from Lake Steinsfjorden, Norway. Environ. Sci. Technol..

[29] Kruger T., Christian B., Luckas B. (2009). Development of an analytical method for the unambiguous structure elucidation of cyclic peptides with special appliance for hepatotoxic desmethylated microcystins. Toxicon.

[30] Diehnelt C.W., Dugan N.R., Peterman S.M., Budde W.L. (2006). Identification of microcystin toxins from a strain of *Microcystis aeruginosa* by liquid chromatography introduction into a hybrid linear ion trap-fourier transform ion cyclotron resonance mass spectrometer. Anal. Chem..

[31] Hummert C., Dahlmann J., Reinhardt K., Dang H., Dang D., Luckas B. (2001). Liquid chromatography-mass spectrometry identification of microcystins in *Microcystis aeruginosa* strain from lake Thanh Cong, Hanoi, Vietnam. Chromatographia.

[32] Yuan M., Namikoshi M., Otsuki A., Sivonen K. (1998). Effect of amino acid side-chain on fragmentation of cyclic peptide ions: Differences of ESI-MS/CID mass spectra of toxic heptapeptide microcystins containing ADMAdda instead of Adda. Eur. J. Mass Spectrom..

[33] Erhard M., von Döhren H., Jungblut P.R. (1999). Rapid identification of the new anabaenopeptin G from *Planktothrix agardhii* HUB 011 using matrix-assisted laser desorption/ionization time-of-flight mass spectrometry. Rapid Commun. Mass Spectrom..

[34] Mayumi T., Kato H., Imanishi S., Kawasaki Y., Hasegawa M., Harada K.-I. (2006). Structural characterization of microcystins by LC/MS/MS under Ion trap conditions. J. Antibiot..

[35] Frias H.V., Mendes M.A., Cardozo K.H.M., Carvalho V.M., Tomazela D., Colepicolo P., Pinto E. (2006). Use of electrospray tandem mass spectrometry for identification of microcystins during a cyanobacterial bloom event. Biochem. Biophys. Res. Commun..

[36] Bateman K.P., Thibault P., Douglas D.J., White R.L. (1995). Mass spectral analyses of microcystins from toxic cyanobacteria using on-line chromatographic and electrophoretic separations. J. Chromatogr. A.

[37] Ferranti P., Fabbrocino S., Nasi A., Caira S., Bruno M., Serpe L., Gallo P. (2009). Liquid chromatography coupled to quadruple time-of-flight tandem mass spectrometry for microcystin analysis in freshwaters: Method performances and characterisation of a novel variant of microcystin-RR. Rapid Commun. Mass Spectrom..

[38] Oksanen I., Jokela J., Fewer D.P., Wahlsten M., Rikkinen J., Sivonen K. (2004). Discovery of rare and highly toxic microcystins from lichen-associated cyanobacterium *Nostoc* sp. strain IO-102-I. Appl. Environ. Microbiol..

[39] Laub J., Henriksen P., Brittain S.M., Wang J., Carmichael W.W., Rinehart K.L., Moestrup Ø. (2002). [ADMAdda^5^]-microcystins in *Planktothrix agardhii* strain PH-123 (cyanobacteria)—Importance for monitoring of microcystins in the environment. Environ. Toxicol..

[40] Yuan M., Namikoshi M., Otsuki A., Rinehart K.L., Sivonen K., Watanabe M.F. (1999). Low-energy collisionally activated decomposition and structural characterization of cyclic heptapeptide microcystins by electrospray ionization mass spectrometry. J. Mass Spectrom..

[41] Sivonen K., Skulberg O.M., Namikoshi M., Evans W.R., Carmichael W.W., Rinehart K.L. (1992). Two methyl ester derivatives of microcystins, cyclic heptapeptide hepatotoxins, isolated from *Anabaena flos-aquae* strain CYA 83/1. Toxicon.

[42] Namikoshi M., Rinehart K.L., Sakai R., Sivonen K., Carmichael W.W. (1990). Structures of three new cyclic heptapeptide hepatotoxins produced by the cyanobacterium (blue-green alga) *Nostoc* sp. strain 152. J. Org. Chem..

[43] Sano T., Kaya K. (1998). Two new (*E*)-2-amino-2-butenoic acid (Dhb)-containing microcystins isolated from *Oscillatoria agardhii*. Tetrahedron.

[44] Sano T., Takagi H., Kaya K. (2004). A Dhb-microcystin from the filamentous cyanobacterium *Planktothrix rubescens*. Phytochemistry.

[45] Sano T., Beattie K.A., Codd G.A., Kaya K. (1998). Two (*Z*)-dehydrobutyrine-containing microcystins from a hepatotoxic bloom of *Oscillatoria agardhii* from Soulseat Loch, Scotland. J. Nat. Prod..

[46] Beattie K.A., Kaya K., Sano T., Codd G.A. (1998). Three dehydrobutyrine-containing microcystins from *Nostoc*. Phytochemistry.

[47] Christiansen G., Yoshida W.Y., Blom J.F., Portmann C., Gademann K., Hemscheidt T., Kurmayer R. (2008). Isolation and structure determination of two microcystins and sequence comparison of the McyABC adenylation domains in *Planktothrix* species. J. Nat. Prod..

[48] Niedermeyer T.H.J., Daily A., Swiatecka-Hagenbruch M., Moscow J.A. (2014). Selectivity and potency of microcystin congeners against OATP1B1 and OATP1B3 expressing cancer cells. PLoS One.

[49] Niedermeyer T.H.J., Schmieder P., Kurmayer R. (2014). Isolation of microcystins from the cyanobacterium *Planktothrix rubescens* strain No80. Nat. Prod. Bioprospect..

[50] Tillett D., Dittmann E., Erhard M., von Döhren H., Börner T., Neilan B.A. (2000). Structural organization of microcystin biosynthesis in *Microcystis aeruginosa* PCC7806: An integrated peptide-polyketide synthetase system. Chem. Biol..

[51] Challis G.L., Ravel J., Townsend C.A. (2000). Predictive, structure-based model of amino acid recognition by nonribosomal peptide synthetase adenylation domains. Chem. Biol..

[52] Sivonen K., Carmichael W.W., Namikoshi M., Rinehart K.L., Dahlem A.M., Niemela S.I. (1990). Isolation and characterization of hepatotoxic microcystin homologs from the filamentous freshwater cyanobacterium *Nostoc* sp. strain 152. Appl. Environ. Microbiol..

[53] Prakash S., Lawton L.A., Edwards C. (2009). Stability of toxigenic *Microcystis* blooms. Harmful Algae.

[54] Kaasalainen U., Jokela J., Fewer D.P., Sivonen K., Rikkinen J. (2009). Microcystin production in the tripartite cyanolichen *Peltigera leucophlebia*. Mol. Plant-Microbe Interact..

[55] Puddick J., Prinsep M.R., Wood S.A., Kaufononga S.A.F., Cary S.C., Hamilton D.P. (2014). High levels of structural diversity observed in microcystins from *Microcystis* CAWBG11 and characterization of six new microcystin congeners. Mar. Drugs.

[56] Marfey P. (1984). Determination of D-amino acids. II. Use of a bifunctional reagent, 1,5-difluoro-2,4-dinitrobenzene. Carlsberg Res. Commun..

[57] Puddick J., Prinsep M.R., Wood S.A., Cary S.C., Hamilton D.P., Wilkins A.L. (2013). Isolation and structure determination of two new hydrophobic microcystins from *Microcystis* sp. (CAWBG11). Phytochem. Lett..

